# Maternal origins and genetic diversity of Sabahan swamp buffalo using mitochondrial cytochrome b gene

**DOI:** 10.1038/s41598-025-18694-4

**Published:** 2025-09-29

**Authors:** Jessica Wyn Brocklebank, Luke Davies, James Hancock, Muralidhar Metta, Kennedy Juani, Jonny Engkias, Benoit Goossens, Pablo Orozco-terWengel

**Affiliations:** 1 School of Biosciences, Cardiff University, Cardiff, UK; 2 Sri Venkateswara Veterinary University, Tirupati, India; 3 Department of Veterinary Services (DVS) Sabah, Level 3, Block B, Wisma Pertanian Sabah, 88624 Kota Kinabalu, Sabah Malaysia; 4 Danau Girang Field Centre, Wisma MUIS, Block B 5th Floor, 88100 Kota Kinabalu, Sabah Malaysia

**Keywords:** Evolution, Evolutionary genetics, Phylogenetics, Population genetics, Agricultural genetics, Evolutionary biology, Haplotypes, Population genetics, Sequencing

## Abstract

The swamp buffalo (*Bubalus bubalis carabanensis*) plays a crucial role in agriculture across Southeast Asia but is increasingly underutilized in regions like Sabah, Malaysia. This study presents the first comprehensive statewide genetic assessment of Sabahan swamp buffalo using mitochondrial cytochrome *b* (*cytb*) gene sequences to investigate maternal origins and assess genetic diversity. Blood samples were collected from 211 individuals across eight locations in Sabah. DNA extraction and PCR amplification successfully yielded 198 high-quality cytb sequences. Comparative analysis with 1745 publicly available sequences revealed that 197 Sabahan buffalo belong to the SA1 haplogroup, which is common across Southeast Asia. Only four haplotypes were identified, with one dominant haplotype found in 190 individuals, indicating remarkably low genetic diversity (haplotype diversity = 0.088; nucleotide diversity = 0.00031). An additional river buffalo haplotype detected in one phenotypically swamp buffalo suggests introgression from Murrah buffalo. Analysis of molecular variance (AMOVA) showed that 96.29% of genetic variation occurred within populations, with minimal differentiation among them. Φ_ST_ comparisons suggest Sabah’s buffalo are genetically closer to Chinese swamp buffalo populations than those from South Asia, supporting a maternal lineage via the China–Taiwan–Philippines dispersal route. Spatial autocorrelation analysis indicated localized gene flow, while demographic reconstruction via Bayesian Skyline Plot suggested long-term population stability with recent expansion. These findings highlight the limited maternal genetic variation within Sabahan swamp buffalo, likely stemming from a founder effect during introduction to Borneo and minimal subsequent genetic inflow. The study provides critical insight into the evolutionary history and population structure of Sabah’s swamp buffalo, with implications for conservation and livestock improvement strategies aimed at bolstering genetic diversity and productivity in the region.

**Supplementary Information:**

The online version contains supplementary material available at 10.1038/s41598-025-18694-4.

## Introduction

The swamp buffalo (*Bubalus bubalis carabanensis*) is one of two domesticated water buffalo species, and is primarily found across Southeast Asia and China where it is famed for its draught capabilities^1,2^. Current evidence suggests that swamp buffaloes were domesticated from the Asian wild water (*Bubalus arnee*) somewhere around northern Thailand^1–6^. Archaeological finds of the earliest swamp buffalo skeletons in this region dates specimens to around 4000 years before present (YBP)^1,7,8^. Similarly, genetic studies estimate a domestication period between 900 and 7000 YBP^5,9^. Current assessment of genetic diversity across the swamp buffalo range consistently shows that genetic diversity is greatest around Thailand, a hallmark of livestock domestication centres^2,5,10–12^. These domestication dates and regions are further corroborated by the belief that swamp buffaloes were domesticated to aid the spread of rice (*Oryza sativa*) cultivation, around 4000–9000 YBP in the Yangzi river basin^13–15^. Post domestication, swamp buffaloes dispersed along two pathways. The first represents a northern route through China during the Shang dynasty, before appearing in Taiwan and the Philippines by 1500 YBP^1,2,7,16^. The second route was a migratory path from mainland Southeast Asia through Sumatra and onto southernly Asian islands^1,2^.

This historic range of Southeast Asia and China is where the majority of the global population of 37 million swamp buffaloes can be found^1,17^. However, despite historically being an incredibly useful livestock species, current trends show population declines^1,17^. Intensification and mechanisation of farming in the last century has led to the redundancy of swamp buffaloes’ draught capabilities, and therefore their replacement^1^. As a result, some areas (e.g., China, Philippines, Australia) are repurposing swamp buffalo for beef production^18,19^. Buffaloes exhibit remarkable adaptability to unforgiving environments and efficiently digest low-quality roughages^20^. Moreover, buffalo meat is notably rich in iron and conjugated linoleic acid (CLA), both of which are essential for maintaining good health^20^. However, raising swamp buffalo for meat production presents certain challenges, as prior selection of swamp buffaloes for short stature, easy handling, resilience, and endurance makes them an excellent draught species, but not highly fruitful for food production^21,22^. Farmers have overcome the low production rates recently by crossbreeding swamp buffalo with the second domestic water buffalo species, the river buffalo (*Bubalus bubalis bubalis*). Domesticated from *B. arnee* in the Indus Valley region around 6000 YBP for milk production, river buffaloes are far larger than swamp buffaloes^1,23^. As such, river buffaloes have been imported into Southeast Asia in attempts to improve traits such as increasing growth and reducing calving intervals in swamp buffalo^22^.

The Malaysian state of Sabah is one location attempting to generate a thriving buffalo industry through new breeding for beef and utilising crossbreeding with river buffalo. Buffaloes in Malaysia are often regarded as important assets but are generally undervalued by farmers as a livestock option, with goats and pigs often preferred^24^. In 2019, the buffalo population in Malaysia was estimated to be around 101,695, split by 46.9% across mainland Malaysia, 46.7% in Sabah, and 6.4% in Sarawak^25^. Again, swamp buffalo numbers are declining and Sabah alone has seen an 11.9% decline within five years prior^25^. The great share of Malaysia’s buffalo population in Sabah is reasoned to be due to the long-established tradition of breeding buffaloes as a supplementary source of income^26^. Buffaloes are often the livestock of choice for the integrated cattle and oil palm farming systems and can often be found throughout community lands across towns and villages^26,27^. The economic status of Malaysia is continuing to increase over time, and in continually developing, an increase in demand for livestock products is often seen in tandem with increased economic status^17^. In 2023, Malaysia imported approximately USD $41.3 million worth of bovine meat, with an annual average of 153 kilo tonnes^28,29^. In comparison, exports of bovine meat were worth USD $2.19 million in 2023, with just 1000 tonnes of beef exported^28–30^. The main sources of imported beef are Australia, India, Brazil and New Zealand, and the high cost of importing beef has contributed to reduced affordability to Malaysians^26^. An improved buffalo industry, with highly productive stock could provide greater benefits for domestic beef production.

This study presents the first statewide genetic analysis of domestic water buffalo in Sabah. Advances in livestock genetics have greatly enhanced productivity through genetic improvement of commercial stocks and identification of unique diversity for conservation^31–34^. As the first phase in the genetic characterisation of Sabahan buffalo, this research focuses on analysing mitochondrial diversity using the cytochrome b (*cytb*) gene. *Cytb* was selected over the D-loop/control region due to its moderate evolutionary rate and reduced susceptibility to homoplasy, which make it more suitable for robust phylogenetic inference and maternal lineage tracing^35^. Furthermore, *cytb* enables broad comparative analysis with existing datasets on swamp buffalo available in public genetic repositories. Mitochondrial DNA analysis was conducted to assess current genetic diversity, trace maternal origins, evaluate potential admixture between swamp and river buffalo, explore the relationship between gene flow and geographic distribution, and reconstruct the demographic history of the population.

## Materials and methods

### Sample collection and DNA extraction

**Fig. 1:**
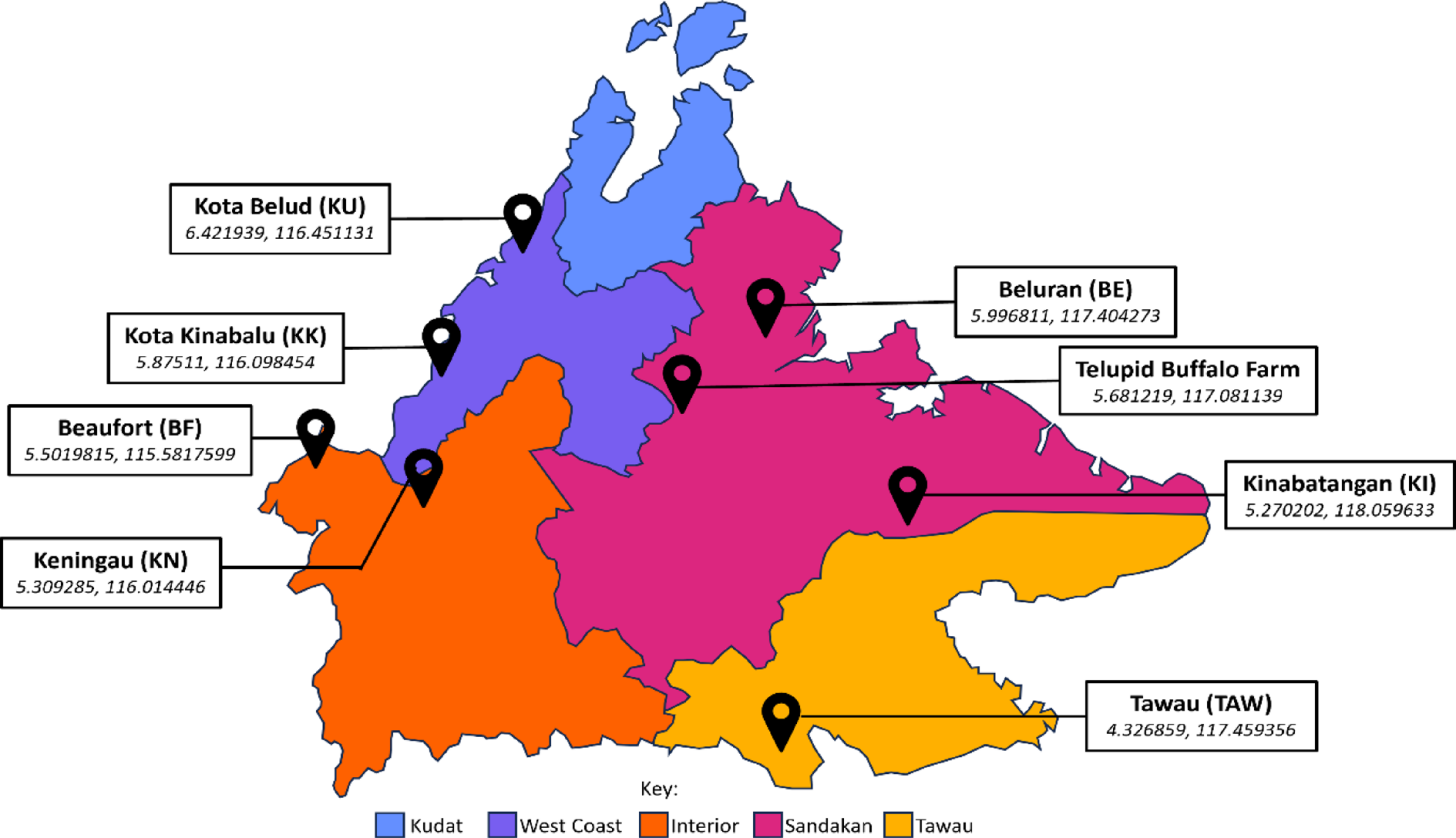
Sabah sampling locations. The five divisions (bahagian) of Sabah coloured, key located at the bottom of the image. Sampling locations indicated by a location pin. Names of sampling locations stated, with their abbreviations in brackets. Italicised are the exact latitude and longitude GPS co-ordinates.

Blood samples were collected from 211 water buffaloes across eight locations of Sabah (Fig. 1). 7 of 8 populations sampled were indigenous swamp buffalo of Sabah. The other one population was a Department of Veterinary Services (DVS) Sabah buffalo research centre. The DVS population was comprised of a variety of swamp buffalo across Sabah, along with some buffaloes imported from Indonesia and Australia, and river-swamp hybrids from imported male Murrah buffalo. 5 ml of blood was extracted from each buffalo via the coccygeal vein and stored in EDTA, which was exclusively undertaken by qualified veterinarians from the Department of Veterinary Services Sabah, approved by Access License granted by the Sabah Biodiversity Council (license reference: JKM/MBS.1000-2/2 JLD.16 (5)). All methods are reported in accordance with ARRIVE guidelines. Of the 211 buffalo sampled, 210 were female and one was male. Among the females, 190 were swamp buffalo and 20 were crossbreeds resulting from swamp buffalo dams and a Murrah river buffalo sire. All crossbreed samples were taken from the DVS Sabah Buffalo Research Centre. The single male was a purebred Murrah. At the DVS Sabah Buffalo Research Centre, 106 buffalo were sampled, of which 24 had been imported from Australia. All buffaloes were adults, although their exact ages were not recorded. DNA was extracted using the Qiagen DNeasy Blood and Tissue Kit, following standard protocol (QIAGEN). DNA was quantified using the Qubit dsDNA Quantification Assay on a Qubit 3 Fluorometer (Invitrogen). A further 1745 publicly available swamp buffalo *cytb* sequences were retrieved from NCBI (FJ467648.1-FJ467917.1, KR009986.1-KR010168.1, MT182026.1-MT182644.1) from previous studies covering mainland Southeast Asia and China^9,36,37^. These were used to aid in determining the maternal origins of Sabahan swamp buffalo and as a comparison for the genetic diversity observed in Sabah.

### PCR and sequencing

Cytochrome b (*cytb*) sequences of 1403 bp were amplified using the primers BBub_CYTB_F (5’-TCAACCACGACCAATGATATGA-3’) and BBub_CYTB_R (5’-AGCTTTGGGTGTTGGTAGTG-3’). PCR reactions consisted of 25ul of 11 µl of 2 × Hot Start *Taq* 2X Master Mix (New England Biolabs), 0.75 µl of 0.25 uM of both BBub_CYTB_F and BBub_CYTB_R, 11 µl of RNAse free water and 1.5 µl of Buffalo DNA were added per PCR reaction. Sterile, RNAse free water was used in leu of buffalo DNA as the negative control. PCR conditions were adapted from Rosli et al.^38^. Initial denaturation took place at 95 °C for 15 min, followed by 35 cycles of: denaturation at 95 °C for 30 s, annealing at 65 °C for 30 s, and extension at 72 °C for 30 s. A final extension at 72 °C for 7 min at the end of the amplification cycles was included. PCR products were sequenced using Sanger sequencing (Eurofins PlateSeq Supreme service). DNA sequence chromatograms were trimmed, and base calls were confirmed by eye in Geneious Prime v2025.0.

### Statistical analysis

Summary statistics of genetic variation (e.g. number of polymorphic sites) were calculated in DnaSP v6.12.03^39^. For the within Sabah analysis of population divergence, the samples were grouped by locality where they were collected (e.g. BE, KK), and the animals of the farm were kept as a separate group. For the between countries comparisons all the samples from Sabah were grouped together and compared to the buffaloes from other countries with their samples grouped by country of provenance. Arlequin v3.5.2.2^40^ was used to carry out a molecular analysis of variance (AMOVA) within Sabah and between countries, as well as to estimate the population pairwise divergence Φ_ST_ . Populations for public data were defined as those used by Sun et al.^37^. Furthermore, groups for AMOVA were set as the geographic regions used by Lei et al.^9^, Zhang et al.^37^ and Sun et al.^36^. The relationship between haplotypes found in Sabah and their relationships to those previously published were assessed with a haplotype network built in PopART v1.7, using the TCS network model^41^. Subsequently, a map of haplotypes distributed across locations was built using the data from PopART.

The demographic history of Sabah’s buffalo population was studied using the Fu and Li’s Fs, and Tajima’s D summary statistics of genetic diversity that are sensitive to demographic changes^42,43^. A BSP analysis was used to model the effective female population size back through time, assuming a relaxed molecular clock to estimate the time of changes in effective population size over time accounting for the potential variation in substitution rate along the sequenced fragment using BEAST v2.7.7.0^44–46^. The model of DNA sequence evolution used was Hasegawa, Kishino, and Yano (HKY) as identified as the best-fit substitution model in MEGA 12 (v.12.0.10)^47,48^. The clock model used was the optimised relaxed clock, with a mean clock rate set to 3.31 × 10^−9^^49^. A total of 100,000,000 steps of the Markov Chain Monte Carlo algorithm were run discarding the first 10% of the steps as burn-in. Model outputs were checked visualised in Tracer (v.1.7.2) by ensuring the effective sample sizes (ESS) of parameters were > 200. BSP traces were plotted using ggplot2^50^.

Spatial autocorrelation in GenAlEx v6.5 was used to determine the relationship between genetic similarity and geographic distance^51^. Pairwise Φ_ST_ calculated in Arlequin was used as the genetic distance, whilst geographic distance (km) was calculated within GenAlEx using latitudes and longitudes of each sampling location. Spatial autocorrelation was assessed for across (i) Sabah populations only, and (ii) across all swamp buffalo populations. Location coordinates for publicly obtained data were taken from their respective studies, and where missing, coordinates were approximated using the centre point of a sampling region^9,36,37^. For both analyses, a test of heterogeneity and correlograms plotting R-values against fixed-distance class intervals were carried out. A fixed-distance class of 150 km was used for the Sabah analysis, and 500 km for all swamp populations. To evaluate the significance of spatial patterns, 1000 bootstrap permutations were performed to generate 95% confidence intervals around r = 0 (depicting the null hypothesis) and to test whether observed R-values significantly differed from expected. Significance was declared when *p* < 0.01^52^.

## Results

### Dataset generation

DNA was successfully extracted for all 211 buffalo samples, with an average yield of 6.49 ng/µl (± 5.36). Furthermore, DNA amplification of *cytb* was successful for all samples. Sample sizes for each location can be found in Table 1. An example of a successful PCR is shown in Supplementary Fig. 1, where a clear band at 1403 bp can be seen following gel electrophoresis of the amplified DNA fragment. After sequencing, 198 of the 211 *cytb* sequences passed quality control. Following trimming of poor-quality sequence ends, a sequence length of 1099 bp was achieved across the Sabahan buffalo dataset. The 198 sequences were uploaded to GenBank and are available under accession numbers PX389714 – PX389911.

**Table 1:** Genetic diversity of *cytb* across Sabahan swamp buffalo populations, and with geographic regions outlined in Sun et al.^3^.

	n	No. haplotypes	No. polymorphic sites	Θ	π
Population					
Beaufort	16	1	0	0.000 ± 0.000	0.00000
Beluran	12	1	0	0.000 ± 0.000	0.00000
Keningau	16	2	1	0.125 ± 0.106	0.00011
Kinabatangan	8	1	0	0.000 ± 0.000	0.00000
Kota Belud	14	1	0	0.000 ± 0.000	0.00000
Kota Kinabalu	18	1	0	0.000 ± 0.000	0.00000
Telupid	74	2	22	0.027 ± 0.026	0.00058
Australia*	22	1	0	0.000 ± 0.000	0.00000
Tawau	18	2	1	0.471 ± 0.082	0.00043
Geographic region					
Sabah	198	4	23	0.088 ± 0.029	0.00031
ML Yangtze	492	30	37	0.529 ± 0.026	0.00331
Upper Yangtze	287	23	33	0.563 ± 0.032	0.00364
SW China and NIC	184	24	44	0.590 ± 0.042	0.00503
SE China	184	24	44	0.590 ± 0.042	0.00503
SE Asia	250	18	33	0.710 ± 0.023	0.00340
South Asia	105	16	47	0.744 ± 0.036	0.01012

Number of haplotypes, polymorphic sites, and diversities calculated in DnaSP (v6.12.03)^9^. Standard deviation shown after ±. * = Individuals originating from Australia but imported to Sabah, found at Telupid as part of government farm breeding programme, n = sample size, Θ = haplotype diversity, π = nucleotide diversity. Abbreviations as follows: ML Yangtze = Middle Lower Yangtze, SW China and NIC = Southwest China and North Indo China, SE China = Southeast China, SE Asia = Southeast Asia.

### Genetic diversity of Sabah swamp buffalo

Four haplotypes were identified across Sabahan buffalo (Table 1). 23 polymorphic sites were found across these haplotypes. Across Sabah, haplotype diversity was 0.088 ± 0.029 (0.000–0.471) and nucleotide diversity was 0.00031 (0.00000–0.00058). The Australian imported buffalo possessed no variation as only one haplotype was found. Totals for each geographic region from the public data were also calculated (Table 1). A detailed breakdown of each of the 56 populations were also calculated (Supplementary Table 1). An AMOVA calculated using just Sabahan swamp buffalo found that 3.71% of genetic variation was accounted for among populations, whilst 96.29% of variation was accounted for within populations (Supplementary Table 2). An AMOVA was repeated on the full swamp buffalo dataset with regional mainland groups (Supplementary Table 3). Variation accounted for between groups was 24.52%. Among populations within each group accounted for 13.79% of variation, and again, as seen in the Sabahan populations alone, variation within populations accounted for 61.69% of all genetic variation.

**Fig. 2:**
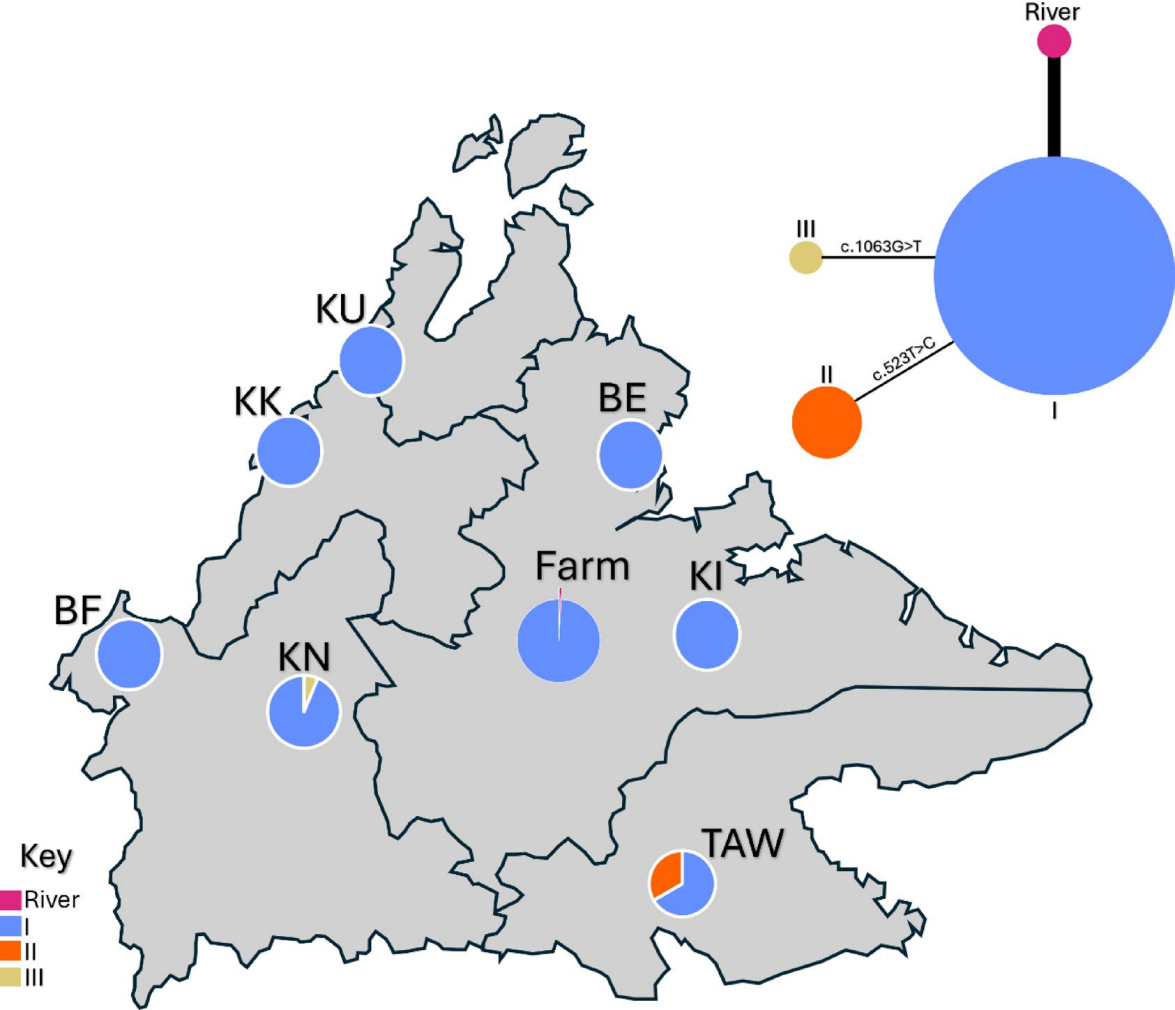
Geographic distribution of haplotypes of Sabahan swamp buffalo. Pie charts calculated using PopART output. Abbreviations as follows; KU = Kota Belud, KK = Kota Kinabalu, BE = Beluran, Farm = Telupid government farm, KI = Kinabatangan, BF = Beaufort, KN = Keningau, TAW = Tawau. Key shown at the left-hand side. Top right panel depicts the *cytb haplotype network of Sabahan swamp buffalo.* I: n = 190, II: n = 6, III: n = 1, River: n = 1. Total number of sequences used = 198. River individual is present in the farm population. Where single mutations separate haplotypes, these have been noted on the thin black line. Thick black line denotes 23 mutations. Haplotype network calculated in PopART.

The haplotype network shows that of the 198 Sabahan swamp buffalo tested, 197 were assigned to the SA1 group that was defined by Wang et al.^5^: I, II and III (Fig. 2). One swamp buffalo (SA_1683) had a river haplotype. This one individual solely accounted for 22 of the 23 polymorphic sites seen. Within the 197 assigned to the SA1, 190 swamp buffaloes were allocated to I, which is the main SA1 haplotype. Six individuals were appointed to II, and one individual was ascribed to III, both with one mutation difference each respectively (Fig. 2). The haplotype map shows low genetic diversity across Sabah as most populations exhibit a dominant haplotype (I) (Fig. 2). However, Tawau and Keningau show some variation, indicated by the presence of additional haplotypes. Telupid also shows an additional haplotype, but this was due to the one river haplotype found in one individual, the other 95 samples were all the I haplotype. All Australian imported samples were of the I haplotype. Counts of haplotypes per population can be found in Supplementary Table 4. The haplotype network of all 1943 sequences can be found in Supplementary Fig. 2.

## Genetic divergence

Between Sabah populations, average Φ_ST_ was 0.049 and ranged from − 0.034 (Telupid vs. Beluran) to 0.278 (Tawau vs. Beaufort) (Supplementary Table 5). Only Tawau consistently showed significant genetic differentiation to other Sabah populations (except Tawau vs. Kinabatangan). Pairwise Φ_ST_ values across all swamp buffalo population comparisons averaged 0.152 and ranged from 0.000 to 0.970 (Supplementary Table 5). Average Φ_ST_ was calculated between regional against Sabah. The results from most similar to most different were as follows: Sabah versus Sabah = 0.049, Sabah versus Upper Yangtze = 0.123, Sabah versus Middle Lower Yangtze = 0.125, Sabah vs Southeast China = 0.127, Sabah versus Southwest China and North Indochina = 0.193, Sabah versus South Asia = 0.700. Sabah populations were most similar to Chinese swamp buffalo regions than Southern Asian populations.

### Isolation by distance

**Fig. 3:**
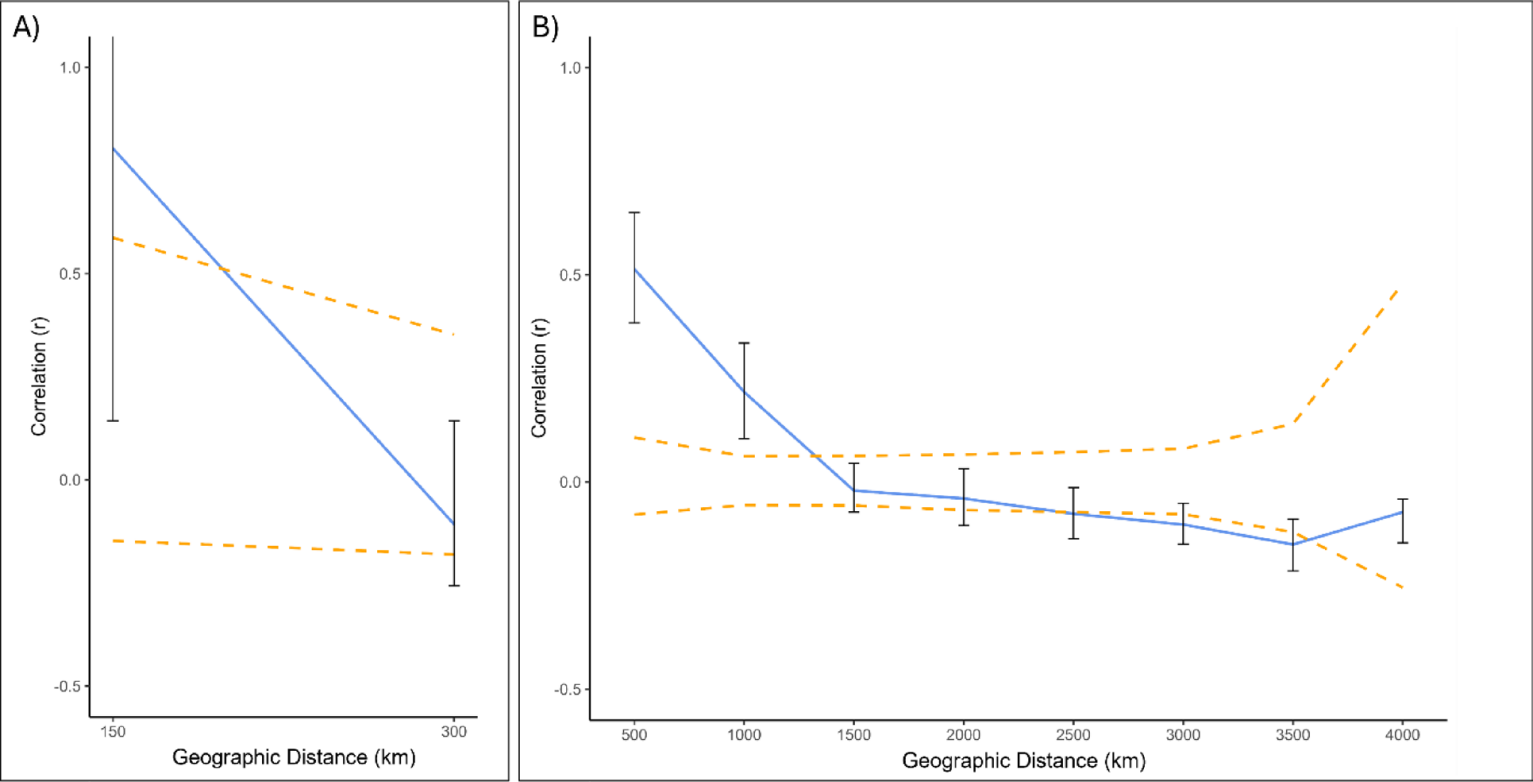
Spatial autocorrelation. Correlograms showing spatial autocorrelation (r) across geographic distance (km) between swamp populations. (**A**) Spatial autocorrelation on Sabahan populations only. Distance class size 150 km. (**B**) All swamp populations. Distance class size 500 km. Dashed lines show the 95% confidence interval surrounding r = 0 indicating no effect of geographic distance on r. 95% confidence error bar is shown for each value of r at each distance.

There was significant spatial autocorrelation across the dataset containing all swamp buffalo populations (Ω = 64.3, *p* < 0.001). This result suggests that there is relation between genetic similarity and geographic proximity. Correlation of haplotypes at short distances begins at r = 0.514 between 0 and 500 km, declining to − 0.021 by 1000–1500 km where r remains stable for the remaining distances (Fig. 3). Analysis of Sabah only populations found non-significant spatial autocorrelation (Ω = 12.3, *p* = 0.032) implying that dispersal within Sabah is not limited at the spatial scale. The Sabah result largely stems from the observation of most swamp buffaloes having the same mitochondrial haplotype (Fig. 2).

### Demographic history

**Fig. 4:**
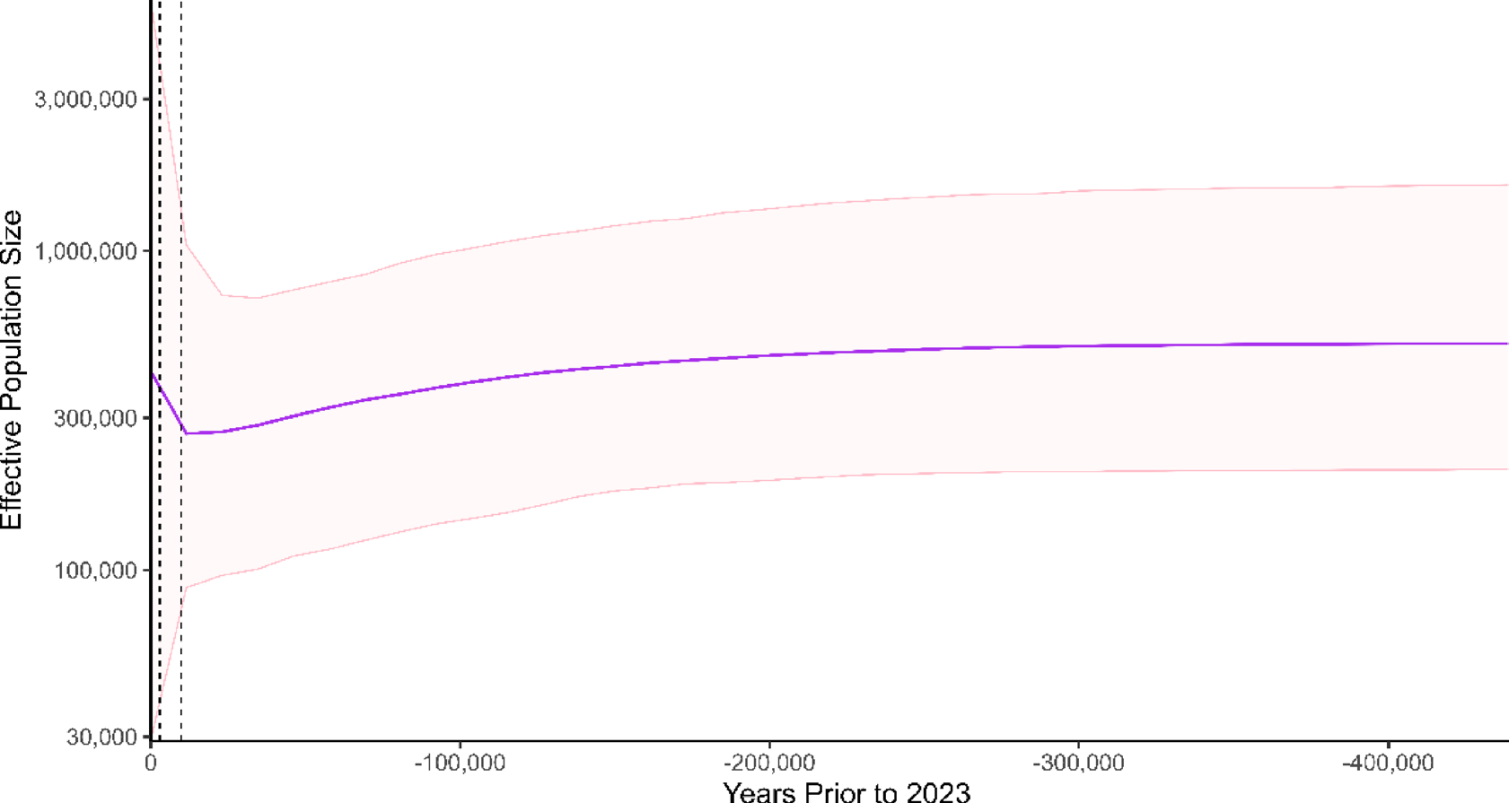
Bayesian skyline plot of Sabahan swamp buffalo. Analysis of 198 Sabahan swamp buffalo *cytb* sequences of 1099 bp length, sampled in 2023 generated in BEAST (v.2.7.7.0) using the Hasegawa-Kishino-Yano (HKY) model with 100,000,000 replicates. X axis in units of years before 2023 CE, Y axis is equal to N_e_ͳ. Clock model was the optimised relaxed clock, and the mean clock rate set to 3.31 × 10^−9^. Purple line is the median estimate, with the pink shaded area denoting the 95% confidence intervals. Dashed black vertical lines indicate the known domestication period of buffalo, 3000–10,000 years ago.

Significant changes in demographics were detected using a variety of statistics in the Sabahan buffalo population. Fu and Li’s Fs was − 1.404, which was not statistically significant to 95% CI. In contrast, Tajima’s D was − 2.53994, with a *p*-value of 0.001 to 95% CI (− 1.32, 2.06). This agrees with previous reports of Chinese and Indochinese buffalo populations^9,53^. Furthermore, BSP shows fluctuations in Sabahan swamp buffalo population size over the last 400,000 years. Between approximately 400,000–250,000 years ago, the population size was relatively stable (Fig. 4). An initial gradual decline in effective population size can be seen from approximately 250,000 years ago to 20,000 years ago, followed by a marked and sharp increase from approximately 20,000 years ago, continuing to 2023. However, wide 95% highest posterior density (HPD) intervals in the more recent past suggest greater uncertainty in recent demographic reconstructions.

## Discussion

This study presents the first statewide genetic analysis of Sabahan buffalo, revealing details surrounding their maternal origins and present genetic diversity. The average haplotype diversity across all Sabah populations was low at 0.088 ± 0.029 (0.000–0.471). This result is much lower than expected when compared to mainland populations, where the average haplotype diversity ranges between 0.267 and 1.00^9,36,37^. Similarly, average nucleotide diversity of 0.00031 found across Sabah is also much lower than seen across mainland populations 0.00075–0.01469^9,36,37^. Low nucleotide diversity in the cytochrome b gene was consistent with a previous study assessing d-loop nucleotide diversity (0.01906) at Sabah’s DVS farm across swamp and crossbred buffaloes^54^. The remarkably low diversity observed in Sabahan buffaloes is concerning for several reasons. First, despite a statewide sampling of buffalo populations, consistently low diversity was found across the dataset. Second, buffaloes from both beef and draught production systems were included, yet low diversity remained evident across these groups. Third, the reduced genetic diversity was not limited to buffaloes of Sabahan origin; it was also observed in animals at the DVS farm in Telupid, that has imported buffaloes from Australia and, previously, Indonesia.

The implications of low genetic diversity in Sabahan buffaloes remain uncertain based on this study. Mitochondrial data provides information from only maternal lineages, lacking insights into paternal lineages^11^. Whilst mitochondrial genomes are not neutral as once thought, the absence of nuclear DNA data limits understanding of genes associated with livestock function that has been integral in livestock genetic improvement^33,55–58^. Nevertheless, some inferences can be drawn. The abundance a single haplogroup, largely represented by a single, dominant haplotype suggests that the present-day mitochondrial diversity seen in the Sabahan buffalo, largely derive from few maternal lineages^59^. The low haplotype diversity likely reflects a historical bottleneck or founder event, wherein a small number of females contributed disproportionately to the gene pool, with this likely occurring through small numbers of buffalo being historically imported to the state^26^. This has been seen in *Ovis canadensis mexicana* in Tiburon Island, where the island population has significantly less genetic variation when compared to mainland populations^60^.

Consequently, these buffaloes may be closely related, and elevated levels of inbreeding could be widespread across the state. Whilst inbreeding is used in livestock to maintain lineages with desirable traits, Sabah lacks formal breeding programmes for swamp buffaloes. Therefore, Sabahan buffaloes may be at greater risk of inbreeding depression if breeding has not been linked to livestock performance and fitness^61–64^. Additionally, reduced genetic diversity restricts fluctuations in allele frequencies over time. As a result, Sabahan swamp buffaloes may face challenges adapting to climate change, such as increased susceptibility to heat stress and related health declines^31^. Meanwhile, between beef production and draught systems, low diversity may prevent Sabahan buffaloes becoming ‘genetically optimised’ for either system.

Sabah is actively seeking to develop its buffalo industry; however, the low genetic diversity observed in Sabahan buffaloes may hinder both production and economic goals. Swamp buffalo are yet to go through substantial selective breeding and genetic improvement for beef production. The limited genetic variation within the population may restrict the potential for selecting and developing improved lineages. Sabahan buffalo may provide the appearance of a ‘rare’ or local breed, whereby the lack of variation makes it difficult to genetically improve without population collapse^32^. A comparable case in Gloucester cattle—a native breed from the United Kingdom—demonstrated that while estimated breeding values could support development, the limited variation in genetic markers reduced the utility of incorporating genomic data^65^. In Sabahan buffalo, a previous study at the DVS farm assessed the heritability of birth weight^66^. The study found that heritability was low (0.29), indicating that other environmental factors affected birth weight, and therefore improvement may be difficult based on genetics alone^66^. Fortunately, Sabahan swamp buffalo are not a distinct breed and as greater diversity is present on mainland Asia, genetic diversity in Sabah could be restored through the importation of unrelated buffaloes from these genetically diverse populations^2,5,9,37^.

The low genetic diversity found across Sabah may have persisted since their arrival to Borneo. It appears that swamp buffaloes were domesticated to aid in rice cultivation as buffaloes excel over other draught animals in wet or waterlogged environments, such as muddy paddy fields^24^. Traditionally, swamp buffalo herds were driven across fields in order to slash weeds and stubble, thus preparing flooded land for rice cultivation^67^. The spread of rice cultivation across southeast Asia corresponds to around 4000 YBP, including in Borneo where evidence has been found in Sarawak^68,69^. However, Borneo does not possess optimal environments for rice production due to its mountainous rainforest environment^70^. It may not have been until rice cultivation methods were established, of which swamp buffalo may have been a part of, that rice and swamp buffaloes spread^70^. Demand for swamp buffalo before this may have been unlikely as, in Borneo, the hunting of bearded pigs (*Sus barbatus*) and banteng (*Bos javanicus*) is embedded in Bornean culture and may have fulfilled this demand for food^71–73^. It has been previously acknowledged that Sabah, and Malaysia as a whole, is lacking in imports of breeding stock to the ruminant industry^26^. Therefore, buffaloes may have only been imported to Borneo along with rice in small numbers, with few subsequent importations.

Following low diversity estimates, four haplotypes were identified across the Sabahan samples. Three of these were swamp haplotypes, all of which are part of the SA1 haplogroup^37^. Unusually, one river haplotype was found within a phenotypically swamp buffalo. Typically, river buffaloes are imported through male lineages, whether importing live individuals or semen. Importation of river buffalo into swamp buffalo regions is common, particularly in the 1980s as the dairy cattle project was introduced^30^. Therefore, identification of a river haplotype indicates that female river buffaloes have been incorporated into crossbreeding practices. This haplotype is readily distinguishable due to the substantial genetic divergence between river and swamp buffaloes. Wang et al.^5^ estimated the divergence between river and swamp mitogenomes to be over 800,000 YBP, while more recent nuclear studies suggest a divergence time of approximately 240,000 YBP. Although both types were domesticated from the wild water buffalo (*Bubalus arnee*), their respective ancestral founders were already genetically distinct^3,5,74^. This prolonged period of separation, coupled with adaptation to different geographic regions (e.g., India versus Southeast Asia), and domestication for different products (milk production versus draught use), has led to considerably different genetic variation between the species^3,74^.

While crossbreeding river and swamp buffaloes may offer a short-term strategy to improve traits such as growth and body size in swamp buffaloes, the profound genetic differences may interfere with established selection patterns and phenotypic traits, potentially resulting in maladaptation to local environmental conditions^31^. If conducted without informed management, such crossbreeding could introduce long-term genetic complications. However, if carefully implemented, the introduction of novel genetic variation from river buffaloes may offer opportunities to develop new, beneficial traits in the Sabahan buffalo population^75^.

190 of samples here were of the primary SA1 haplotype. The other two swamp haplotypes were of a single mutation difference to this primary haplotype and found uniquely associated with single populations of Tawau and Keningau and are not found within the mainland populations. As such, Φ_ST_ results within Sabah populations showed there was low genetic differentiation between populations. Tawau was the only Sabahan population that differed significantly to the others. This is likely due to the additional II haplotype that is present only in this population, at a greater proportion than that of III in Keningau. Approximately half of the Sabahan buffalo sampled in this study derived from one population, being the DVS farm (Telupid). Local Sabah populations were assumed to comprise of buffalo local to Sabah, with no influence from imported genetics since buffaloes are bred locally. Meanwhile, the DVS farm contains a mixture of genetics from Sabah, Australia, and previous Indonesian imports. Therefore, it would be expected that this population does not reflect those of local Sabahan buffalo populations. If genetic improvement efforts at the DVS farm are successful, livestock at the DVS farm may become more desirable and valuable owing to greater productivity and economic output. This could promote the distribution of these improved animals across the state, potentially leading to the replacement of local genetic lineages by improved and imported genetics which could in turn have consequences of conservation of genetic resources in livestock. However, the impact of this replacement would be undetectable using mitochondrial data alone, as many of these animals share identical haplotypes. Therefore, further research using nuclear markers is essential to accurately assess the genetic diversity and structure of both local and farmed buffalo populations.

As the SA1 haplogroup was found in Sabah, it is difficult to confirm the origins of Sabahan buffalo because this haplogroup is prevalent across the swamp buffalo range. However, Φ_ST_ analysis here shows that it is more likely that Sabahan buffalo originated via the Chinese-Taiwan-Philippines pathway. Φ_ST_ scores were consistently lower verses Chinese populations than Southernly mainland Asia populations. This is due to the absence of rare haplogroups present in Chinese populations as only SA and SB haplogroups were captured in historical migrations^37^. Additional uptake of new haplotypes was not possible in China, as the only extant buffalo species at the time was *Bubalus mephistopheles,* which doesn’t appear to have been crossed with swamp buffalo^76^. Most notably, the population in Taiwan is almost completely comprised of SA1 haplogroup which may explain why *cytb* diversity is low in Sabah. Each migration away from a domestication centre captures a smaller proportion of genetic variation^11,12^. As such, rarer haplogroups were likely lost into China, and further diversity lost into Taiwan. This indicates that the Philippines and Sabah may only have a small pool of variation to capture. A previous study found that Malaysian buffalo tended to not cluster with Thai buffalo, further supporting a Chinese origin^35^. However, patterns of variation in Sabah may not be representative of wider Borneo, as Indonesian buffaloes possess different D-loop haplotypes in the north and south^77^.

The analysis of spatial autocorrelation across all swamp buffalo revealed that there was greater gene flow occurring at shorter distances (< 1000 km). Such relationship suggests that swamp buffalo are likely to breed locally or with nearby populations as, for example, local people exchange livestock in markets, and that animals tend to not be exchanged at greater distances^53^. Within the Sabah only analysis, potential high correlation is observed at the first distance (0–150 km), before becoming uncorrelated at 150–300 km. Mitochondrial diversity in Sabahan buffaloes is dominated by a single haplotype, therefore this result is unsurprising. The drop in correlation may be driven by Tawau, a population that is more distant compared to other sites and features another haplotype in abundance. AMOVA results may further support population structure results as most of the variation was assigned to within populations rather than among populations and groups. Whilst we may infer that Sabahan buffaloes derive from a Chinese migratory pathway, the generation of nuclear data would give greater resolution into understanding their relationship with mainland populations^55^.

Interestingly, within our samples collected were Australian imported swamp buffaloes. These are imported into Sabah to the DVS Buffalo Research Farm that is the Telupid population here. These individuals show improved beef production capabilities, thus aiding to improve native stock. All Australian individuals also showed an absence of diversity, as all were assigned into the SA1 haplogroup. Swamp buffalo were imported to Australia in the early nineteenth century from Timor and surrounding islands^78,79^. How these individuals effect Sabahan buffaloes genetic diversity is unclear from this study, however nuclear DNA from southern Indonesian islands would suggest Australian buffalo possess genetic diversity originating from the Thai-Sumatra migration pathway, therefore bringing a new influx of genetic diversity to Sabah^2^. These low haplotype and nucleotide diversities for each individual population, and the Australia subset of Telupid could be due to sampling bias, however, as there is little haplotype diversity across the whole state, this may indicate that it is not sampling bias^80^.

A BSP analysis was used to model the effective female population size back through time. Results largely reflected those of wider swamp buffalo studies, despite the smaller amount of data available here^5,81^. Swamp effective population size continually declined towards the domestication period (< 10,000 YBP). This may be due to overhunting of wild water buffalo prior to domestication, restricting animal resources for ancient people that triggered improved animal management leading to domestication^82^. Effective population size increases from that point, throughout the domestication period, and to the present day. In buffalo, this may be explained by accumulation of new haplotypes through uptake of new wild female buffaloes over time, or otherwise new mutations over time. This is well known in river buffalo where hybridisation with wild water buffalo is still occurring in the present day^1,23,83^. Results from statistics evaluating demographic changes (e.g., Tajima’s D) support a population expansion. Fu and Li’s D* and F* along with Tajima’s D all returned significant negative values indicating an excess of uncommon haplotypes^9,84^. Previous studies across swamp buffalo have showed that population expansion have occurred, such as in Chinese swamp buffalo^9,53^. However, Fu’s Fs is considered the most sensitive statistic and was the only non-significant result^42^. Therefore, it may be considered that Sabah’s buffalo population is relatively stable.

Overall, this study found that swamp buffalo deriving from Sabah, Borneo possessed remarkably low diversity across the state, dominated by a single haplogroup. The identification of a river buffalo haplotype within a swamp buffalo indicates that there has been greater admixture besides importation of a few males or imported semen. The lack of other swamp haplogroups found suggests that Sabahan buffalo derive from the China-Taiwan-Philippines migration route as opposed to Thailand-Sumatra that possesses a greater variety of haplogroups. Our findings provide valuable insights into the evolutionary history and genetic structure of Sabahan swamp buffalo, contributing to conservation strategies and the sustainable management of this important livestock species.

## Supplementary Information

Supplementary Material 1

## Data Availability

All datasets generated and analysed in study have been deposited in GenBank and are accessible under accession numbers PX389714 – PX389911.
