# Correction: Maternal origins and genetic diversity of Sabahan swamp buffalo using mitochondrial cytochrome b gene

**DOI:** 10.1038/s41598-026-36191-0

**Published:** 2026-01-15

**Authors:** Jessica Wyn Brocklebank, Luke Davies, James Hancock, Muralidhar Metta, Kennedy Juani, Jonny Engkias, Benoit Goossens, Pablo Orozco-terWengel

**Affiliations:** 1 School of Biosciences, Cardiff University, Cardiff, UK; 2 Sri Venkateswara Veterinary University, Tirupati, India; 3 Department of Veterinary Services (DVS) Sabah, Level 3, Block B, Wisma Pertanian Sabah, 88624 Kota Kinabalu, Sabah Malaysia; 4 Danau Girang Field Centre, Wisma MUIS, Block B 5th Floor, 88100 Kota Kinabalu, Sabah Malaysia

Correction to: *Scientific Reports* 10.1038/s41598-025-18694-4, published online 29 September 2025

The original version of this Article contained an error, where the GenBank accession numbers were omitted.

As a result, in the Results section, under the subheading ‘Dataset generation’,

“Following trimming of poor-quality sequence ends, a sequence length of 1099 bp was achieved across the Sabahan buffalo dataset.”

now reads:

“Following trimming of poor-quality sequence ends, a sequence length of 1099 bp was achieved across the Sabahan buffalo dataset. The 198 sequences were uploaded to GenBank and are available under accession numbers PX389714 – PX389911.”

In addition, in the Data availability section,

“The datasets generated and/or analysed during the current study are not publicly available as the data forms part of an ongoing study and is currently being used for additional analyses. Data is available from the corresponding author on reasonable request.”

now reads:

“All datasets generated and analysed in study have been deposited in GenBank and are accessible under accession numbers PX389714 – PX389911.”

The original Article has been corrected.

